# Editorial: Modulating Cortical Dynamics in Language, Speech and Music

**DOI:** 10.3389/fnint.2018.00058

**Published:** 2018-11-27

**Authors:** Gesa Hartwigsen, Mathias Scharinger, Daniela Sammler

**Affiliations:** ^1^Research Group Modulation of Language Networks, Department of Neuropsychology, Max Planck Institute for Human Cognitive and Brain Sciences, Leipzig, Germany; ^2^Phonetics Research Group, Department of German Linguistics, Center for Mind, Brain and Behavior, Philipps-Universität Marburg, Marburg, Germany; ^3^Otto Hahn Group “Neural Bases of Intonation in Speech and Music”, Max Planck Institute for Human Cognitive and Brain Sciences, Leipzig, Germany

**Keywords:** non-invasive brain stimulation (NIBS), transcranial magnetic stimulation (TMS), transcranial direct current stimulation, network, virtual lesion, cognition

Language, speech and music are uniquely human channels of communication resting on evolved neurocognitive circuits. Research on the neurobiological foundations of these abilities has taken considerable strides across the past 25 years. Accordingly, current large-scale models provide fine-grained maps of specialized fronto-temporo-parietal networks that support complementary computational goals. Dorsal and ventral stream models for speech and language (Hickok and Poeppel, 2007; Rauschecker and Scott, 2009; Friederici, 2011; Bornkessel-Schlesewsky and Schlesewsky, 2013), vocal pitch (Sammler et al., 2015), and music (Loui, 2015; Peretz, 2016), and sensorimotor control models of speech (Guenther and Hickok, 2015; Houde and Chang, 2015) and song (Berkowska and Dalla Bella, 2009; Zarate, 2013) are just a few examples.

Based on these models, new questions on the functional *dynamics* within and across these large-scale networks arise. For instance, it remains largely unclear how key regions operate and communicate with each other, from lower (sensory) to higher (cognitive) levels. Another central question concerns the *functional relevance* of these regions to specific processes and their differential contribution across individuals. Recently, macro-anatomical lesion studies and correlative neuroimaging approaches have been complemented by non-invasive brain stimulation (NIBS) methods that promise answers to these questions. Through *focal* modulation of neural activity, they provide a means to directly probe the causal contribution of circumscribed cortical regions to a given task (Pascual-Leone et al., 1999; Walsh and Cowey, 2000; Kuo and Nitsche, 2012) and allow for an investigation of adaptive network dynamics on the systems level (Hartwigsen, 2018).

This Research Topic comprises a collection of one review and five original research papers that applied state-of-the-art transcranial magnetic stimulation (TMS) and transcranial direct-current stimulation (tDCS) to increase our understanding of the cortical dynamics within and between the large-scale neural networks underlying language, speech, and music in the healthy brain. Together, they reveal the dynamic exchange between hemispheres (Andoh et al.; Hohmann et al.), the temporal dynamics within brain regions (Zhang et al.), as well as the specific division of labor within networks (Ishibashi et al.). Moreover, they show individual differences in network dynamics (Andoh et al.) with potential consequences for behavior and neuromodulatory effects of NIBS (Deroche et al.; Schaal et al.).

Starting at the level of auditory cortex, Andoh et al. provide a comprehensive review of NIBS studies that shed light on local and remote interactions in auditory areas contributing to speech and music perception. Specifically, this review highlights functional differences of left and right auditory cortex and argues for strong, yet asymmetric interhemispheric interactions between auditory regions that largely depend on individual connectivity patterns. Providing a translational link, the authors also discuss the therapeutic potential of NIBS in the treatment of auditory neurological disorders such as tinnitus.

Moving from auditory perception to production, three studies used tDCS and TMS to investigate causal contributions of dorsal stream regions in the production of speech or vocal pitch. Focusing on speech motor learning, Deroche et al. applied tDCS to left inferior parietal lobe (IPL) to modulate motor adaptation to altered auditory feedback. Participants exhibited increased adaptation under facilitatory (anodal) tDCS, showing that tDCS of left IPL can enhance speech motor learning. No effects of anodal tDCS were found in conditions with unaltered feedback, arguing for a specific role of left IPL in learning, but not regular speech motor control. This is in line with the notion of a strong context-dependency of NIBS effects (Silvanto and Cattaneo, 2017). Focusing on vocal pitch production, Hohmann et al. found decreased performance in targeting and fine-tuning vocal pitch in a humming task after inhibition of right posterior superior temporal gyrus (STG) and left posterior inferior frontal gyrus (IFG) by means of cathodal tDCS. They propose specific roles of these areas in feedback and feed-forward motor control of pitched vocal production. These results provide additional support for the relevance of interhemispheric interactions (see also Andoh et al.). Zhang et al. further zoomed into the temporal dynamics of left IFG involvement in speech production using chronometric TMS. They applied triple pulse TMS at different time points during picture naming in Mandarin Chinese speakers, and found strongest delays in response times when TMS was applied as early as 225 ms after picture onset. These results are taken to suggest that phonological encoding in Mandarin Chinese occurs a little earlier than in Indo-European languages (Indefrey and Levelt, 2004), highlighting language-dependent inter-individual differences.

Finally, two studies used tDCS and TMS to elucidate higher-level cognitive functions in language and music. Ishibashi et al. dissociated the specific roles of left anterior temporal lobe (ATL) and IPL in semantic cognition. Anodal tDCS of the left ATL improved access to knowledge about both function and manipulation of common tools, while stimulation of IPL had selective effects on function knowledge only, supporting the hub-and-spokes model of semantic representations (Ralph et al., 2017). In turn, Schaal et al. show causal involvement of right dorsolateral prefrontal cortex in pitch memory and highlight the dependency of NIBS effects on individual baseline abilities. Accordingly, cathodal tDCS selectively impaired performance of non-musicians in a pitch span task, but only if they had a high baseline pitch memory. These results stress inter-individual variability in response to neuromodulatory effects of NIBS protocols (Hamada et al., 2013) that may emerge from different network configurations and dynamics between individuals.

Together, these studies emphasize the value of NIBS to investigate causal structure-function relationships, and modulate cognitive dynamics in the language, speech and music domains. These findings pave the way for future applications in basic research and therapeutic settings. The way forward will include multi-method combinations of NIBS and electrophysiological or neuroimaging techniques to provide a comprehensive characterization of the functional relevance and interaction of specific regions within and between the neural networks for language, speech, and music, as well as their individual dynamics and differences.

## Author contributions

All authors listed have made a substantial, direct and intellectual contribution to the work, and approved it for publication.

### Conflict of interest statement

The authors declare that the research was conducted in the absence of any commercial or financial relationships that could be construed as a potential conflict of interest.
